# Diverse Control Mechanisms of the Interleukin-1 Cytokine Family

**DOI:** 10.3389/fcell.2022.910983

**Published:** 2022-06-27

**Authors:** Charles L. Evavold, Jonathan C. Kagan

**Affiliations:** ^1^ Ragon Institute of MGH, MIT and Harvard, Cambridge, MA, United States; ^2^ Division of Gastroenterology, Boston Children’s Hospital and Harvard Medical School, Boston, MA, United States

**Keywords:** IL-1, inflammasomes, pyroptosis, hyperactivation, gasdermin D, secretion, cytokines, inflammation

## Abstract

The majority of interleukin-1 (IL-1) family cytokines lack amino terminal secretion signals or transmembrane domains for secretion along the conventional biosynthetic pathway. Yet, these factors must be translocated from the cytoplasm across the plasma membrane into the extracellular space in order to regulate inflammation. Recent work has identified an array of mechanisms by which IL-1 family cytokines can be released into the extracellular space, with supramolecular organizing centers known as inflammasomes serving as dominant drivers of this process. In this review, we discuss current knowledge of the mechanisms of IL-1 family cytokine synthesis, processing, and release from cells. Using this knowledge, we propose a model whereby host metabolic state dictates the route of IL-1β secretion, with implications for microbial infection and sterile inflammation.

## Introduction

Production and secretion of interleukin-1 (IL-1) family cytokines is closely linked to inflammation. All IL-1 family cytokines, except IL-1Ra, lack an amino terminal (N-terminal) secretion signal for secretion by the endoplasmic reticulum (ER)-Golgi vesicular pathway (Garlanda et al., 2013). Several family members, such as IL-1α, IL-1β, and IL-36α/β/γ, are considered pro-inflammatory. Other members, such as IL-1Ra and IL-36Ra, serve inhibitory or buffering roles that counteract the pro-inflammatory functions of IL-1α/β and IL-36 cytokine signaling, respectively. Select IL-1 family cytokines can also serve anti-inflammatory functions in the case of IL-37 and IL-38 or context-dependent pro-inflammatory and anti-inflammatory functions in the case of IL-18 and IL-33.

IL-1α and IL-1β (sometimes referred to in aggregate as IL-1) have related functions within the host through action on their shared heterodimeric receptor IL-1R1 and IL-1R accessory protein known as IL-1R3 (Mosley et al., 1987a; Mosley et al., 1987b; Sims et al., 1988; Greenfeder et al., 1995). Through cloning of pro-IL-1β, it was readily appreciated that this inactive precursor molecule did not contain an N terminal signal sequence highlighting a major conundrum on how the bioactive form of this cytokine might exit the cell to act on its cognate receptor (Auron et al., 1984; Rubartelli et al., 1990). The IL-1 receptor complex, when ligated to IL-1α or IL-1β, but not when ligated to the inhibitory protein IL-1Ra, can recruit the signaling adaptor MyD88 (Wesche et al., 1997). MyD88 recruitment and its downstream pro-inflammatory signaling events are similar to the sensing of pathogen associated molecular patterns (PAMPs) by pattern recognition receptors (PRRs) of the Toll-like receptor (TLR) family. As such, many of the pro-inflammatory functions of TLRs are recapitulated by IL-1 family receptors. A major action of IL-1R signaling is the activation of the transcription factor NF-κB leading to production of pro-inflammatory cytokines, upregulation of antigen presentation, and pro-survival signaling in various cell types (O'Neill, 2008). In addition, IL-1R signaling can provide mitogenic signals in the case of T and B lymphocytes, as reviewed elsewhere (Evavold and Kagan, 2018). Recent work has also highlighted that IL-1 signaling can induce an antiviral state in fibroblasts (Orzalli et al., 2018; Aarreberg et al., 2019).

Signaling through other IL-1 receptors appears to follow analogous processes to IL-1R, whereby the cognate ligand of an IL-1 family cytokine binds a heterodimeric receptor that induces the recruitment and activation of MyD88 (Garlanda et al., 2013; O'Neill, 2008). The anti-inflammatory action of some IL-1 family members may stem from differential usage of MyD88 for pro-inflammatory versus anti-inflammatory responses. For example, IL-33 binding to the specific IL-33 receptor known as IL-1R4 (also known as ST2) can be considered pro-inflammatory on type 2 T helper (Th2) cells and mast cells (Ali et al., 2007; Chackerian et al., 2007). IL-33 bound IL-1R4 can then recruit the accessory protein IL-1R3, as is the case for the IL-1 receptor complex, to recruit and activate MyD88 (Garlanda et al., 2013). Conversely, IL-33 signaling on T regulatory cells (Tregs) can be considered anti-inflammatory through induction of proliferation of this inherently anti-inflammatory cell type and production of the tissue repair factor known as amphiregulin (Arpaia et al., 2015; Kuswanto et al., 2016). Analogous to TLR contextual signaling, TLR4 and TLR5 expressing Tregs also appear to use TLR-MyD88-dependent signaling for anti-inflammatory and tissue repair related responses (Caramalho et al., 2003; Crellin et al., 2005).

Thus, as IL-1 family cytokines can have location and cell-type-dependent responses, leading to either the induction or resolution of inflammation, this family of cytokines is under increased regulation compared to conventionally secreted counterparts. Regulation of the induction, maturation, and secretion of these cytokines is the focus of this review.

## Overview of IL-1 Family Cytokines

As stated above, IL-1 is the prototypical member of the IL-1 family of cytokines. IL-1 acts on many cell types to induce inflammation including, but not limited to, endothelial cells, epithelial cells, myeloid cells, and lymphocytes (Evavold and Kagan, 2018). IL-1 can also trigger the secretion of additional conventional cytokines and chemokines, such as IL-6 and IL-8 respectively, that promote local inflammation through increasing the permeability of endothelial cells for immune cell recruitment and systemic inflammation through induction and maintenance of fever and production of acute phase proteins in the liver (Garlanda et al., 2013).

**FIGURE 1:**
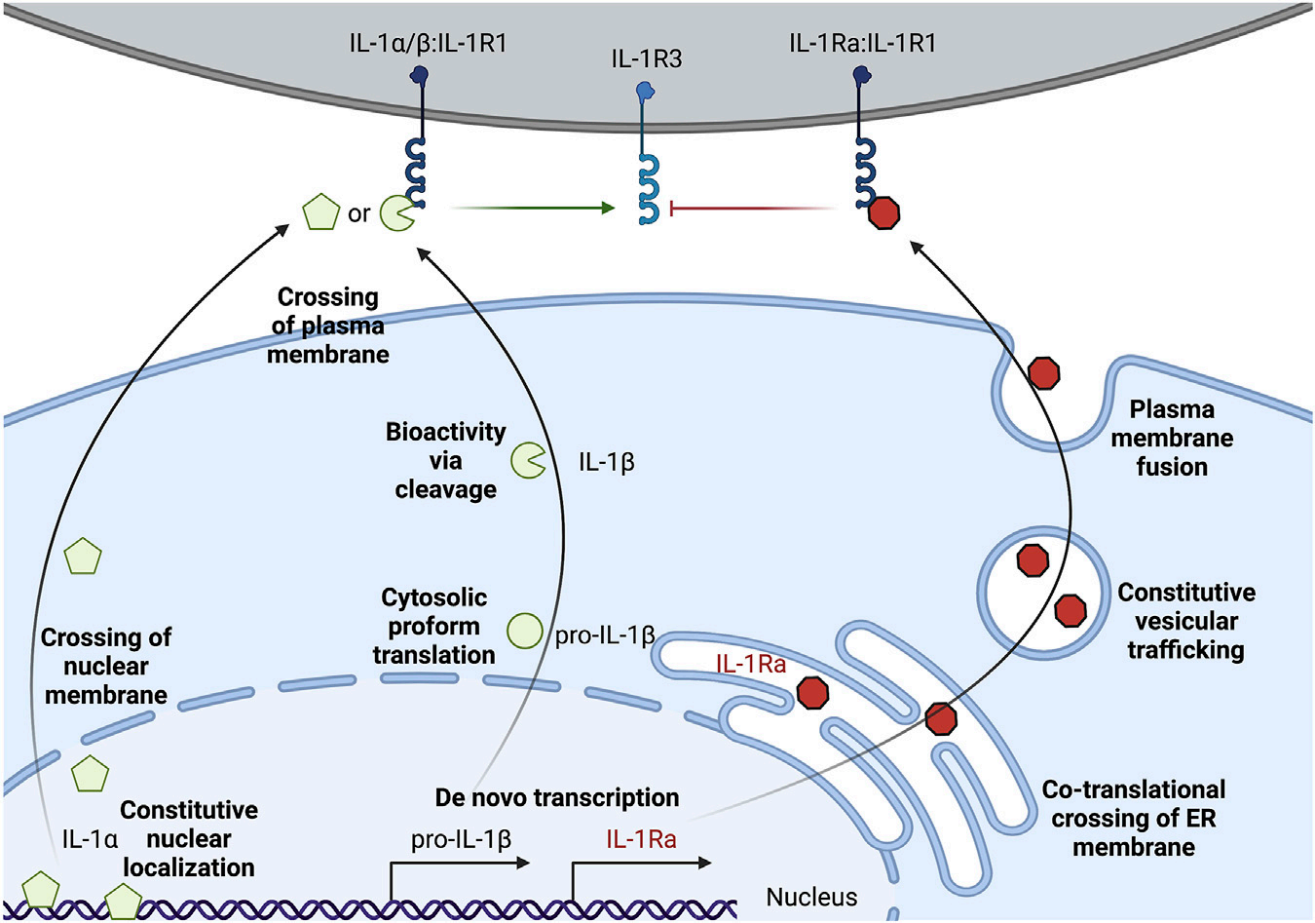
Multi-level regulation of unconventional secretion of IL-1. IL-1α can be constitutively expressed, but accumulates in the nuclear compartment. Secretion requires crossing the topological barriers of the nuclear membrane and the plasma membrane. Proform IL-1α is biologically active on the IL-1 receptor complex, but cleavage by certain proteases such as calpains can increase activity. Pro-IL-1β is often transcriptionally induced upon sensation of lower level threats to the host such as extracellular PAMPs or pro-inflammatory cytokines. Pro-IL-1β is translated and remains in the cytosol, and must cross the topological barrier of the plasma membrane for secretion. Cleavage of pro-IL-1β by proteases such as caspase-1 is required for bioactivity on the IL-1 receptor complex. IL-1Ra is the only IL-1 family member that is conventionally secreted through the biosynthetic pathway. IL-1Ra is transcriptionally induced alongside sensation of inflammatory cues and conventional secretion of this cytokine may buffer the action of pro-inflammatory IL-1α/IL-1β by blocking their interaction with IL-1R1.

**FIGURE 2:**
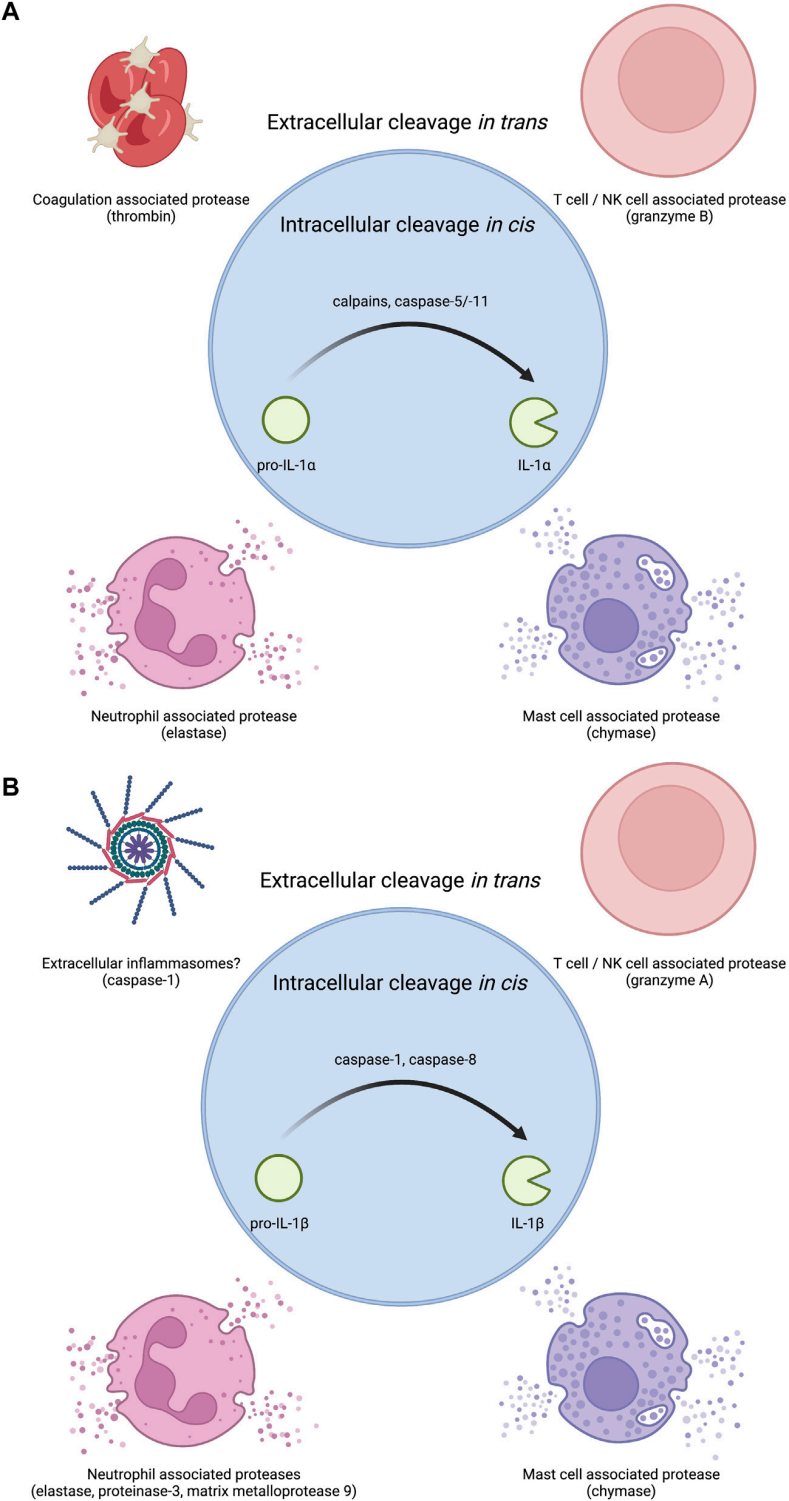
Proteases that regulate IL-1 bioactivity *in cis* or *in trans*. **(A)** The precursor protein for IL-1α is inherently bioactive. Several proteases have been shown to increase this bioactivity. Intracellular proteases that can regulate IL-1α *in cis* include calcium activated proteases such as calpains as well as the non-canonical inflammasome associated caspase-5 and caspase-11 in human and mouse respectively. Extracellular proteases that can regulate IL-1α *in trans* include coagulation associated thrombin, T cell/NK cell associated granzyme B, neutrophil associated elastase, and mast cell associated chymase. **(B)** Pro-IL-1β must be processed into IL-1β to become bioactive. Several proteases have been shown to mediate this conversion to bioactivity. Intracellular proteases that can regulate IL-1β *in cis* include inflammasome associated caspase-1 or diverse complexes that can contain caspase-8. Extracellular proteases that can regulate IL-1β *in trans* include T cell/NK cell associated granzyme A, neutrophil associated proteases elastase, proteinase-3, and matrix metalloprotease 9, and mast cell associated chymase. As inflammasome specks can exist in the extracellular space after pyroptotic lysis, extracellular inflammasomes may also be capable of regulating extracellular pro-IL-1β cleavage likely through caspase-1.

IL-1α exists as a pro-form cytokine primarily within the nucleus of cells (Werman et al., 2004; Lamacchia et al., 2013). Some cell types, such as epithelial cells, appear to constitutively express IL-1α, though pro-inflammatory signaling can induce the production of new pools of IL-1α. The subcellular localization of this cytokine is attributed to a nuclear localization signal (NLS) within the pro-domain (Werman et al., 2004; Wessendorf et al., 1993). IL-1α is best known for its pro-inflammatory activities resulting from ligation and activation of the IL-1 receptor complex. This necessitates that IL-1α egresses the nucleus and makes it to the extracellular space to act on IL-1 receptor complexes on other cells (Figure 1). While pro-form IL-1α can signal through the IL-1 receptor complex (Kim et al., 2013), the potency of IL-1α on its cognate receptor increases after processing by select proteases (Figure 2). Examples of such proteases include calpains, which are calcium-dependent cysteine proteases located at the inner leaflet of the plasma membrane (Kobayashi et al., 1990; Afonina et al., 2011). Thus, while IL-1α can be released upon cellular necrosis, the activity of IL-1α is increased following regulated secretion that includes disruption of the nucleus and calcium (Ca) flux (Gross et al., 2012; England et al., 2014). These events occur during certain cell death processes such as induction of pyroptosis through the action of inflammasomes, which will be discussed in the following sections (Keller et al., 2008; Gross et al., 2012). Recent studies indicate that IL-1α can also be released from cells after sublytic inflammasome stimulations and from living cells, such as occurs during phagocyte hyperactivation or early pyroptotic stimulations (Gardner et al., 2015; Evavold et al., 2018; Tapia et al., 2019; Wiggins et al., 2019; Aizawa et al., 2020; Tsuchiya et al., 2021). While the canonical inflammasome component caspase-1 can mediate the calpain-dependent processing and subsequent release of IL-1α (Gross et al., 2012; Tsuchiya et al., 2021), caspase-1 is unable to directly process pro-IL-1α (Howard et al., 1991). In contrast, recent work has identified that inflammatory caspase-5/-11 can directly process IL-1α into a more bioactive molecule (Wiggins et al., 2019). The increased bioactivity of processed IL-1α can also be contextually controlled *in trans*. Under these circumstances, a necrotic cell may release pro-form IL-1α that is then cleaved by proteases from a different cell, such as mast cell chymase, neutrophil elastase, or cytolytic T-lymphocyte (CTL) and natural killer (NK) cell granzyme B (Lüthi et al., 2009; Clancy et al., 2018). Moreover, IL-1α can be activated after cleavage by the coagulation cascade associated protease thrombin (Burzynski et al., 2019) (Figure 2).

IL-1β is generally associated with myeloid lineage cells such as macrophages, dendritic cells, and neutrophils (Chan and Schroder, 2020). In their resting (non-inflammatory state), these myeloid cells do not express pro-form IL-1β and typically require a pro-inflammatory signal to initiate transcription and translation, such as after TLR activation upon microbial encounters (Figure 1). Conversely, certain cell types, such as keratinocytes, may constitutively express low levels of IL-1β without pro-inflammatory stimuli (Mizutani et al., 1991a). Pro-IL-1β is found within the cytosol (Chan and Schroder, 2020). This pro-form cytokine requires proteolytic processing to become biologically active on the IL-1 receptor complex (Howard et al., 1991; Thornberry et al., 1992) (Figure 2). This cytokine also must be released from the cytosol into the extracellular space to reach IL-1 receptor complexes on other cells (Figure 1). Thus, in contrast to IL-1α, which must cross the nuclear and plasma membrane to access the extracellular space, cytosolic IL-1β must only traverse the plasma membrane. The lower threshold of crossing the membrane of a single compartment for IL-1β to escape the cell might be explained through the additional regulation at the induction of transcription, compared to a pre-existing pool of nuclear IL-1α in some cell types. Moreover, unlike IL-1α, IL-1β has an absolute requirement for its cleavage to achieve bioactivity. Thus, multiple strategies of IL-1 regulation appear to mediate IL-1α and IL-1β release from cells.

The cleavage and release of IL-1β is often closely linked to the action of inflammasomes (Evavold and Kagan, 2019; Chan and Schroder, 2020). Inflammatory caspase-1 cleaves pro-IL-1β into mature IL-1β (Kostura et al., 1989; Thornberry et al., 1992; Li et al., 1995). Caspase-1 also cleaves the protein gasdermin D (GSDMD) to mediate pyroptotic lysis of cells (He et al., 2015; Kayagaki et al., 2015; Shi et al., 2015). GSDMD cleavage by caspase-1 releases a fragment that oligomerizes into pores in host cell membranes (Aglietti et al., 2016; Liu et al., 2016a; Ding et al., 2016; Sborgi et al., 2016). GSDMD pores can mediate IL-1β release in direct and indirect ways (Evavold et al., 2018; Heilig et al., 2018). Moreover, inflammatory caspases-4, -5, -11 can also mediate the cleavage and secretion of IL-1β, but this process requires the secondary activation of the NLRP3 inflammasome and caspase-1 for direct IL-1β cleavage (Kayagaki et al., 2015; Rühl and Broz, 2015; Shi et al., 2015). The necroptotic pathway can also cause membrane permeability and rupture *via* the pore forming protein MLKL and pro-IL-1β processing *via* the NLRP3 inflammasome (Gutierrez et al., 2017). Recent work has also illustrated that caspase-8 can mediate the cleavage and release of IL-1β in contexts where traditional inflammasome components are lacking or under conditions of TAK1 inhibition (Orning et al., 2018; Sarhan et al., 2018; Muendlein et al., 2020). Finally, pro-IL-1β can be cleaved in the extracellular space *in trans via* mast cell-associated chymase, neutrophil-associated elastase, proteinase-3, matrix metalloprotease 9, and CTL and NK cell-associated granzyme A (Black et al., 1988; Hazuda et al., 1990; Mizutani et al., 1991b; Coeshott et al., 1999) (Figure 2).

IL-1Ra is an inhibitory protein to the IL-1 receptor complex (Arend et al., 1989; Arend et al., 1994). IL-1Ra is the only IL-1 family member that contains an N-terminal signal sequence for translation at the endoplasmic reticulum, trafficking through the Golgi, and fusion and release at the plasma membrane (Figure 1) (Garlanda et al., 2013). The highly inflammatory nature of IL-1α and IL-1β on cells expressing the IL-1 receptor complex may explain why this inhibitory member of the IL-1 family evolved to be conventionally secreted. IL-1Ra binds to the same IL-1R1 as IL-1α and IL-1β, thus limiting the pro-inflammatory signaling that these cytokines induce (Arend et al., 1989; Arend et al., 1994). IL-1Ra bound IL-1R1 cannot productively signal through IL-1R3. This may serve as a local and systemic buffering system to limit low levels of autoinflammation during constitutive death processes or during resolution of inflammation. Other cytokine systems, such as the conventional cytokine IL-6, also have mechanisms to buffer the signaling propensity of the pro-inflammatory cytokine *via* the production of secreted decoy receptors (Yousif et al., 2021). In addition to IL-1Ra-dependent inhibition of IL-1R1 signaling, IL-1 is also scavenged by a membrane bound and soluble decoy receptor known as IL-1R2 (Colotta et al., 1993; Re et al., 1996; Kuhn et al., 2007; Lorenzen et al., 2012). These two strategies in addition to the cell-intrinsic and *in trans* regulation of IL-1α and IL-1β cytokine processing and release illustrate that these cytokines are highly inflammatory. Indeed, several autoinflammatory diseases, such as cryopyrin-associated periodic syndrome (CAPS) and familial Mediterranean fevers (FMF), and autoimmunity diseases, such as rheumatoid arthritis (RA) and multiple sclerosis (MS), are associated with overproduction and secretion of IL-1 (Garlanda et al., 2013). Recombinant IL-1Ra (known as Anakinra) is used as a therapy in some of these indications, and monoclonal antibodies against IL-1β (such as Canakinumab) demonstrate similar reduction in inflammation associated with neutralizing the bioactivity of this cytokine (Dinarello, 2018).

IL-18 is expressed constitutively in certain cell types such as epithelial and myeloid cells (Puren et al., 1999). Similar to IL-1β, IL-18 is an inactive, pro-form cytokine produced in the cytosol of cells (Okamura et al., 1995; Ghayur et al., 1997). IL-18 is cleaved into a bioactive cytokine *via* inflammasome activated caspase-1 (Ghayur et al., 1997; Gu et al., 1997). IL-18 can also be directly cleaved by inflammatory caspase-4 (Kobayashi et al., 2013; Knodler et al., 2014). This contrasts with the indirect role of caspase-4 in activating the NLRP3 inflammasome for IL-1β processing *via* caspase-1 (Kayagaki et al., 2015; Rühl and Broz, 2015). Notably, these molecular themes of IL-1 cleavage apply to humans and mice, but not all mammals. A subset of carnivores (excluding canines) can utilize a hybrid inflammatory caspase to detect bacterial cell wall lipopolysaccharides (LPS), akin to human caspase-4, and also mediate IL-1β cleavage directly (Devant et al., 2021). As such, this hybrid enzyme, known as caspase-1/4, operates as a one-protein signaling pathway that bypasses the need for an inflammasome and directly links LPS detection to IL-1β cleavage in an analogous manner to human caspase-4 direct cleavage of IL-18.

The specific IL-18 receptor is known as IL-1R5 (formerly IL-18 receptor α chain). When IL-1R5 binds cleaved IL-18, IL-1R5 recruits the signaling competent accessory protein known as IL-1R7 (formerly known as IL-18 receptor β chain) (Dinarello, 2018). Downstream signaling occurs through recruitment of MyD88, as is the case for the activated IL-1 receptor complex. IL-18 signaling can be considered pro-inflammatory as it can mediate inflammation *via* immune cell recruitment to tissues and upregulation of antigen presentation (Garlanda et al., 2013). IL-18 also functions to impact adaptive immunity in concert with conventionally secreted pro-inflammatory cytokines from myeloid cells, such as IL-12 and IL-15 (Okamura et al., 1995; Evavold and Kagan, 2018). The original name for IL-18 was IFN-γ inducing factor because IL-18 in combination with IL-12 (or IL-15) can instruct T lymphocytes to differentiate towards the *Th1* helper subset, and thus encourage IFN-γ production *via Th1* and NK cell lymphocytes (Okamura et al., 1995; Ghayur et al., 1997). Consistent with this pro-inflammatory role of IL-18, several autoinflammatory and autoimmune diseases are associated with increased serum concentrations of IL-18 including CAPS, FMF, and MS (Garlanda et al., 2013). Unlike IL-1, IL-18 does not induce fevers when administered exogenously (Gatti et al., 2002). While IL-18 has been purported to have protective roles in colitis, subsequent work suggests that IL-18 mediates inflammation and epithelial barrier dysfunction (Nowarski et al., 2015). Similar to the buffering activity of the membrane-bound decoy receptor IL-1R2 towards the bioactivity of IL-1, the host produces a conventionally secreted protein called IL-18 binding protein (IL-18bp) to scavenge IL-18 and likely dampen the inflammatory activities of IL-18 (Novick et al., 1999). While more studies are needed to delineate the magnitude and kinetics of production of receptor antagonists, binding proteins, and decoy receptors for other IL-1 family members, IL-18bp is well characterized as a buffering system for IL-18 driven inflammation (Novick et al., 1999; Kim et al., 2000; Novick et al., 2001). During homeostasis, serum concentrations of IL-18bp seem to be constitutively higher than serum concentrations of IL-18 by at least an order of magnitude (Novick et al., 2001). As IL-18bp can bind in a 1 to 1 M fashion to IL-18 with tight affinity, this means that at baseline even homeostatic production of IL-18 is buffered or chelated by IL-18bp (Novick et al., 1999; Kim et al., 2000; Novick et al., 2001). During inflammation, IL-18 levels must surmount the levels of IL-18bp to mediate bioactivity on the cognate cytokine receptor. Interestingly, IL-18bp is upregulated by IFN-γ during inflammation (Mühl et al., 2000; Hurgin et al., 2002). As IL-18 can induce the production of IFN-γ as mentioned above (Okamura et al., 1995; Ghayur et al., 1997), this transcriptional feedback loop may initiate resolution of inflammation unless high levels of IL-18 continue to be produced. Other factors that affect the IL-18 to IL-18bp setpoint require further characterization, but this example illustrates that IL-1 family members are under additional extracellular regulation likely to limit inappropriate inflammation at baseline and promote return to homeostasis quickly following resolution of a pathogenic insult.

Similar to IL-1α, IL-33 is expressed as a nuclear pro-form cytokine that has inherent bioactivity when released from cells in an unprocessed form (Carriere et al., 2007; Talabot-Ayer et al., 2009; Bessa et al., 2014). Thus, as is the case in IL-1α regulation, the presence of an NLS and pro-domain act as two barriers to IL-33-mediated inflammation. In contrast to IL-1β and IL-18, caspase-1 processing of IL-33 may abrogate bioactivity (Cayrol and Girard, 2009). Similarly processing by apoptotic executioner caspases such as caspase-3/-7 also leads to diminished bioactivity (Lüthi et al., 2009). It is unknown whether this processing can occur within the nucleus during cell death programs or whether it primarily occurs as the nuclear compartment is damaged and IL-33 egresses through the cytosol on its way to the extracellular space. Moreover, inflammasomes have been shown to exist in inflamed tissues apart from the initial source pyroptotic cell (Baroja-Mazo et al., 2014; Franklin et al., 2014). While it is unknown how much caspase-1 activity might be retained within these extracellular “ASC specks,” the presence of relatively few pyroptotic events may have effects on the bioactivity of IL-33 released from other cells in a local tissue environment. These data suggest that IL-33 can be a contextual signal for caspase-independent necrotic or necroptotic cell death processes (Lüthi et al., 2009; Ohno et al., 2009). IL-33 can be processed *in trans* by proteases, such as mast cell-associated chymase and neutrophil elastase, that increase bioactivity (Bae et al., 2012; Lefrançais et al., 2012; Waern et al., 2013; Roy et al., 2014). IL-33 binds to the specific receptor known as IL-1R4 (formerly ST2) to mediate recruitment of the signaling competent accessory protein IL-1R3 that is also used by the IL-1 receptor complex and IL-18 receptor complex (Ali et al., 2007; Chackerian et al., 2007). The activated IL-33 receptor complex can then recruit MyD88 to activate NF-κB-dependent processes (Garlanda et al., 2013). IL-33 can incur pro-inflammatory functions through activation and proliferation of the *Th2* helper subset of T lymphocytes in contexts such as multicellular parasite infection or allergy (Ali et al., 2007; Bartemes et al., 2012; Garlanda et al., 2013). IL-33 can act as an anti-inflammatory cytokine through proliferation and upregulation of the tissue repair cytokine amphiregulin in T regulatory cells in contexts such as muscle injury (Arpaia et al., 2015; Kuswanto et al., 2016).

The IL-36 subfamily consists of IL-36α, IL-36β, IL-36γ, IL-36Ra, and IL-38 (Dinarello, 2018). These members all bind to the specific IL-36 receptor chain known as IL-1R6 (formerly known as IL-1 receptor-related protein 2) (Towne et al., 2004). The production and response to IL-36 cytokines primarily occurs at barrier sites such as the squamous epithelium of the skin (Boutet et al., 2016). Keratinocytes transcribe and translate IL-36γ after sensation of PAMPs such as poly (I:C) and flagellin (Lian et al., 2012). IL-36γ is released after poly (I:C) treatment of keratinocytes in a caspase-3/-7-dependent manner that also requires upstream caspase-1 activation (Lian et al., 2012). While little is known regarding the processing and secretory mechanism of IL-36 members, the association with inflammasome related caspase-1 and apoptotic caspase-3/-7 may suggest that the gasdermin family of pore forming molecules may play a role in secretion of IL-36 from keratinocytes as is the case for IL-1β and IL-18. While recombinant full-length IL-36 cytokines can elicit bioactivity, N-terminally truncated IL-36 increases bioactivity on the IL-36 receptor complex (Towne et al., 2011). The IL-36 cytokines do not have obvious caspase cleavage motifs, but there may be distinct proteases that cleave IL-36 either in a secreting cell or *in trans* as is the case for other IL-1 family members. This might proceed through either caspase-1-dependent GSDMD pore formation or caspase-3/-7-dependent GSDME pore formation. IL-36 can signal to epithelial cells, such as skin keratinocytes, to produce chemokines that may mediate inflammation through recruitment of immune cells to the site of IL-36 release (Li et al., 2014). Moreover, IL-36 is produced in lesions associated with the autoimmune disorder psoriasis (Johnston et al., 2011; Marrakchi et al., 2011). The inhibitory protein IL-36Ra inhibits IL-36 receptor signaling by blocking binding of the activating ligands IL-36α, IL-36β, and IL-36γ to IL-1R6 in an analogous way to IL-1Ra action on the IL-1 receptor complex (Dinarello, 2018). Deficiency in IL-36Ra is associated with pustular psoriatic lesions in the skin, again highlighting that beyond IL-36 processing and release that additional regulation at the level of receptor binding is required to prevent autoinflammation and autoimmunity for inflammatory IL-1 family members (Blumberg et al., 2007; Marrakchi et al., 2011; Sugiura et al., 2014). IL-38 is a partial antagonist of IL-36-dependent inflammation as IL-38 binds the same IL-36 receptor complex as agonist IL-36 cytokines (van de Veerdonk et al., 2012). IL-38 has an anti-inflammatory role as it can block IL-22 and IL-17A production in response to *Candida albicans* (van de Veerdonk et al., 2012; Han et al., 2019). As IL-38 is elevated in patients with the autoinflammation such as asthma and autoimmune diseases such as SLE and RA, this cytokine may act similarly to IL-1Ra, IL-18bp, and IL-36Ra in buffering the inflammatory actions of agonist IL-36 cytokines (Rudloff et al., 2015; Boutet et al., 2016; Chu et al., 2016). The potential role of processing and mechanisms of IL-38 release await further characterization.

IL-37 is another IL-1 family member that is transcriptionally regulated and sequestered to the nucleus until programmed release into the extracellular space where it can exert anti-inflammatory functions (Sharma et al., 2008; Nold et al., 2010). IL-37 is expressed in epithelial cells, lymphocytes, and myeloid cells (Dinarello, 2018). Mice do not express an orthologue of human IL-37, but ectopic expression in mice and murine cells demonstrates anti-inflammatory properties (Nold et al., 2010). IL-37 binds to IL-1R5 which is the same specific ligand receptor for IL-18 signaling (Kumar et al., 2002; Nold-Petry et al., 2015). In contrast to IL-18/IL-1R5 recruitment of IL-1R7 for IL-18 signaling, IL-37 binding to IL-1R5 recruits the chain IL-1R8 (also known as SIGIRR) (Li et al., 2015; Nold-Petry et al., 2015). IL-37 is released in both a cleaved and unprocessed form after inflammasome activation in human myeloid cells, but processing is not necessary for bioactivity of IL-37 (Kumar et al., 2002; Bulau et al., 2014; Li et al., 2015). Release of IL-37 after inflammasome signaling may serve to mitigate or buffer the inflammatory potential of inflammasome released IL-1 or other sources of inflammation due to the presence of microbial ligands in an infected tissue. One model that has been proposed for how IL-37 could be anti-inflammatory is through sequestering MyD88 to the TIR domain of IL-1R8, thus depriving other TLR and pro-inflammatory IL-1 family receptors of their required signaling adaptor (Gong et al., 2010). This intracellular buffering of pro-inflammatory signaling again highlights the potency of IL-1 family members and the requirement for multiple levels of regulation to their inflammatory actions. As several members of the IL-1 family seem to utilize contextual processing and release *via* the inducible organelles known as inflammasomes, we will provide updates on regulation of inflammasome signaling and membrane permeabilization in subsequent sections.

## Regulation of Inflammasomes

Inflammasomes are threat-assessing organelles that assemble in response to cytosolic perturbations indicative of pathogen invasion or sterile damage (Evavold and Kagan, 2019; Chan and Schroder, 2020). Inflammasomes have many layers of regulation that affect the cleavage and secretion of bioactive IL-1 family cytokines. This regulation can take the form of transcriptional control of inflammasome components and substrates, post-translational control of location and conformation of inflammasome components, and control of negative regulators of inflammasome signaling. Inflammasomes consist of a seed protein, oligomerization unit, and enzymatic effector (Evavold and Kagan, 2019). Many intracellular PRRs have been determined to serve as seed proteins for inflammasome activation, including proteins of the NLR family, the protein Pyrin, the protein AIM2, and the recently discovered protein CARD8.

Some inflammasomes require a two-signal integration of threat level for optimal activation (Evavold and Kagan, 2019; Chan and Schroder, 2020). The two-signal requirement of certain inflammasomes serves as a logic gate to prevent the inappropriate release of bioactive IL-1 and inflammatory cell death. This logic gate is best exemplified by the synergistic recognition of microbial ligands by the TLR family and subsequent activation of the NLRP3 inflammasome in myeloid cells, such as macrophages. Unstimulated macrophages do not express appreciable amounts of the inflammasome seed protein NLRP3 or pro-IL-1β. Only upon PRR detection of PAMPs, such as TLR4 sensing bacterial LPS, or through the action of certain pro-inflammatory cytokines, such as TNFR sensing TNFα, can an NF-κB-dependent transcriptional response upregulate NLRP3 and pro-IL-1β. Thus, low-level threats of extracellular microbial ligands, stress ligands, or pro-inflammatory cytokines can poise a sentinel cell to survey for the presence of higher threats such as pathogen invasion of the cytosol or manipulation of host machinery. This transcriptional upregulation of inflammasome components and inflammasome substrates has been termed “priming” or “signal one.” Other inflammasomes such as the AIM2 inflammasome and the caspase-11 inflammasome are under the control of a transcriptional signal one, though these receptors typically require the induction of an interferon (IFN) response for their transcriptional upregulation. Beyond the upregulation of transcriptional responses, priming can also post-translationally modify inflammasome proteins or alter lipid organization on membranous organelles to mediate conformational changes or subcellular location of inflammasome proteins.

The second signal in inflammasome activation is the trigger for seed oligomerization. This process is intrinsically controlled by receptor location because all known inflammasome receptors are located within the cytosol (or nucleus) and are thus topologically separated from low-level threats, such as microbial ligands in the extracellular space (Evavold and Kagan, 2019). A higher threat, such as microbial ligands in the sterile cytosol or dysfunction of a cellular process, are thus used as indications of pathogen invasion and result in a commensurate inflammatory response of release of bioactive IL-1 and in some cases lytic cell death.

Inflammasomes consist of several distinct seed proteins that can sense diverse inputs, but triggering of these receptors converge on oligomerization of adaptor ASC (and in some cases NLRC4) to promote the activation of inflammatory caspase-1. ASC is recruited to most inflammasome seeds, such as NLRP3, NLRP6, AIM2, and Pyrin, through PYRIN-PYRIN domain interactions. The NAIP proteins sense proteins structurally related to components of bacterial secretion or motility machinery to recruit the adaptor NLRC4. The oligomerization of the adaptors ASC and NLRC4 in the above inflammasomes serves to recruit pro-caspase-1 and induce activation of caspase-1 through enforced proximity. Oligomers of NLRC4 or ASC recruit pro-caspase-1 through CARD-CARD domain interactions. Increasing the local concentration of caspase-1 within the inflammasome filament allows for pro-caspase-1 and various caspase-1 heterodimers to process other caspase-1 molecules *in trans* at two linker locations (Thornberry et al., 1992; Boucher et al., 2018). These cleavage events cause the formation of distinct species of active caspase-1 heterodimers including an inflammasome localized, highly active species consisting of a p33 and p10 fragment and a solubilized species consisting of a p20 and p10 fragment (Boucher et al., 2018). This sequential cleavage illustrates tight regulation on the duration and magnitude of caspase-1 activity within cells that may be intrinsically related to the size or available oligomerization surfaces of inflammasome assemblies (Boucher et al., 2018; Evavold and Kagan, 2019).

## Role of IL-1 Cleavage in Bioactivity, Membrane Localization, and Secretion

Caspase-1 activity is intimately related to the cleavage of intracellular substrates such as the select IL-1 family members IL-1β, IL-18, IL-33, and IL-37 (Chan and Schroder, 2020). As stated above, inflammasome associated caspase-1 can cleave IL-1β, IL-18, and IL-37 to increase their binding and bioactivity to their respective cytokine receptors (Thornberry et al., 1992; Ghayur et al., 1997; Gu et al., 1997; Kumar et al., 2002). In the case of IL-33, caspase-1 may process the cytokine into a moiety that is no longer bioactive (Cayrol and Girard, 2009). In the context of IL-1β and IL-18, cleavage of pro-form cytokine can change the overall isoelectric point of the protein (Monteleone et al., 2018). The pro-domain of IL-1β is negatively charged, whereas the polypeptide corresponding to the mature p17 fragment is positively charged. Thus, cleavage of pro-IL-1β into IL-1β releases an overall positively charged mature cytokine that becomes enriched in the inner leaflet of the plasma membrane through charge-charge interactions with negatively charged phospholipid headgroups, such as PI(4,5)P2 (Monteleone et al., 2018). Accumulation of IL-1β at the plasma membrane can facilitate fast release through GSDMD pores or slow release by underdetermined mechanisms. Caspase-1 also facilitates the secretion of bioactive IL-1 family cytokines through regulation of the pore forming protein GSDMD (Kayagaki et al., 2015; Shi et al., 2015; Evavold et al., 2018; Heilig et al., 2018).

GSDMD pores are recognized to be size and charge-dependent conduits for the secretion of IL-1 from hyperactivating and sublytic inflammasome stimulations (Evavold et al., 2018; Heilig et al., 2018; Xia et al., 2021). The structure of the human GSDMD pore was recently determined through cryo-EM of lipid nanodisk containing oligomerized N-terminal fragments of GSDMD (Xia et al., 2021). Through charge reversal point mutations in the context of GSDMD and the cargo mature IL-1β, it was determined using liposome release assays and sublytic inflammasome stimulations in reconstituted murine macrophages that GSDMD allows the enriched release of mature IL-1β through electrostatic filtering (Xia et al., 2021). This appears to primarily operate through repulsion of negatively charged pro-IL-1β from the pore channel as opposed to selective preference for mature IL-1β.

## Regulation of GSDMD Pores

All gasdermin family members, except Pejvakin, contain an N-terminal domain that can form a plasma membrane pore (Ding et al., 2016). As such, the gasdermin family has been the subject of recent investigation of unconventional protein secretion, membrane permeability, and cell death. GSDMD exists as a latent protein within the cytosol of resting cells (Kayagaki et al., 2015; Shi et al., 2015). Upon inflammasome activation, GSDMD is cleaved in a flexible linker region that contains a caspase cleavage site (Liu et al., 2020; Wang et al., 2020). Inflammatory caspases (e.g., caspase-1/-4/-5/-11) recognize GSDMD *via* an exosite in the C terminal fragment (Liu et al., 2020; Wang et al., 2020). Caspase-8 can also cleave GSDMD—possibly during death receptor signaling, alternative inflammasome activation, TAK1 inhibition, or during *Yersinia* infection (Gaidt et al., 2016; Orning et al., 2018; Sarhan et al., 2018; Donado et al., 2020). While caspase-8 can also be recruited and activated on ASC assemblies of canonical inflammasomes (Sagulenko et al., 2013; Vajjhala et al., 2015), this may primarily occur in contexts where pyroptosis is delayed or defective, such as genetic deficiencies in caspase-1 and GSDMD (Schneider et al., 2017; Tsuchiya et al., 2019).

The primary role of inflammatory caspases and caspase-8 in activating GSDMD is releasing the pore forming N terminal fragment from the auto-inhibitory C terminal domain (Ding et al., 2016; Liu et al., 2019). However, in certain contexts such as gut inflammation, full length GSDMD may mediate the unconventional secretion of IL-1β (Bulek et al., 2020). This study did not see robust cleavage of GSDMD by immunoblot assay but noted a genetic requirement of GSDMD for IL-1β release. Other studies have found that sublytic stimulations, such as infections with mutant *S. aureus*, may cleave GSDMD for IL-1β secretion below the limit of detection by immunoblot (Evavold et al., 2018; Bjanes et al., 2021). Thus, determining whether full length gasdermins might truly form membrane pores awaits further characterization—though there is evidence that point mutations in GSDMD at the binding interface between the N and C terminus can relieve autoinhibition and cause membrane binding and pore formation by the full-length protein (Liu et al., 2019). Either the removal of an inhibitory post-translational modification or addition of an activating modification may alter GSDMD pore formation through the function of the C-terminal autoinhibitory domain, the accessibility of the caspase cleavage site, or membrane binding and oligomerization potential of the N-terminal domain. Indeed, GSDMD was recently described to be modified by host metabolites at cysteine residues in the N-terminus (Humphries et al., 2020; Bambouskova et al., 2021). These modifications appear to block the cleavage of full length GSDMD by inflammatory caspases thus limiting GSDMD oligomerization and pore formation (Humphries et al., 2020; Bambouskova et al., 2021). Of note, one of these cysteine residues has also been implicated in oligomerization of a GSDMD pore after cleavage (Liu et al., 2016a; Rathkey et al., 2018; Hu et al., 2020; Humphries et al., 2020). Use of non-specific cysteine modifying agents, such as necrosulfanamide and disulfiram, can covalently modify cysteine 192 that may sterically hinder the ability of GSDMD N-terminal fragments to oligomerize (Rathkey et al., 2018; Hu et al., 2020). Moreover, the change of the corresponding cysteine to alanine or more conservatively to serine can impact oligomerization and cell death in 293T cells (Liu et al., 2016a; Hu et al., 2020; Humphries et al., 2020). Recent work from our group has determined that reactive oxygen species (ROS) metabolites can enhance GSDMD pore formation that requires cysteine 192 (Devant et al., 2022). More work is required to delineate the role of post-translational modifications (PTMs) in regulating gasdermin function.

The N-terminal fragment of GSDMD has affinity for negatively charged phospholipids such as phosphatidylserine and PI(4,5)P2 found in the inner leaflet of the plasma membrane (Liu et al., 2016a; Ding et al., 2016). Furthermore, GSDMD can bind to other negatively charged lipids, such as cardiolipin, that is present in bacterial or mitochondrial membranes (Aglietti et al., 2016; Liu et al., 2016a; Ding et al., 2016; Sborgi et al., 2016). GSDMD mediates lysis of bacteria after intracellular expression or treatment of liquid cultures and has recently been shown to target mitochondria in the context of pyroptosis (Liu et al., 2016a; Ding et al., 2016). How GSDMD accesses cardiolipin, which is normally found on the inner membranes of intact mitochondria and is topologically hidden by the bacterial cell wall, has not been determined. Cardiolipin becomes externalized after stress (Iyer et al., 2013; Elliott et al., 2018), so GSDMD may target damaged or stressed mitochondria and bacteria.

At the plasma membrane, GSDMD pores can mediate calcium flux from the hypercalcemic extracellular space into the hypocalcemic cytosol (Martín-Sánchez et al., 2016; Russo et al., 2016; Rühl et al., 2018). As IL-1β and GSDMD both localize to PI(4,5)P2-containing regions of the plasma membrane (Liu et al., 2016a; Monteleone et al., 2018), the formation of a GSDMD pore may allow a transient release of calcium that promotes removal of membrane enriched IL-1β through the action of PLC-γ cleavage of PI(4,5)P2 into DAG. During this transient removal of IL-1β from the membrane, PI(4,5)P2 metabolism by PLC may also mediate conformational changes of the pore to limit the amount of IL-1β that is released (Santa Cruz Garcia et al., 2022). Sustained calcium flux is a trigger for membrane repair processes, such as lysosomal exocytosis or ESCRTIII-dependent membrane blebbing, to remove compromised sections of the plasma membrane (Cooper and McNeil, 2015; Rühl et al., 2018). Moreover, this calcium flux mediates the rapid conformational opening or closing of GSDMD through a mechanism that may involve metabolism of phospholipids [e.g., PI3K formation of PI(4,5)P2 and PI(3,4,5)P3 or degradation of these species to DAG by calcium-dependent phospholipases such as PLC-γ] (Santa Cruz Garcia et al., 2022). Therefore, ion flux and lipid metabolism may regulate GSDMD pore formation and the rate of secretion of mature IL-1β.

Downstream of GSDMD cleavage, the Ragulator-Rag protein complex, which controls mTOR signaling, is required for GSDMD oligomerization and pore formation (Evavold et al., 2021). The role of Ragulator-Rag in GSDMD regulation was linked to the production of ROS metabolites, which are necessary to promote GSDMD oligomer formation and pyroptosis (Evavold et al., 2021). How ROS metabolites affect GSDMD pore formation is still unknown, but they could directly affect GSDMD cysteine residues either through addition of activating PTMs or removal of inactivating PTMs. In support of this model, our recent work has determined that oxidation state of cysteines in the N terminus of GSDMD are drastically different in RagA-deficient cells that do not form pores compared to wild type macrophages (Devant et al., 2022). Moreover, defects in GSDMD pore formation in RagA-deficient cells could be rescued through diverse ROS inducers (Devant et al., 2022). Beyond these indications that ROS may directly regulate GSDMD oligomerization within living cells, ROS may mediate additional indirect effects on related cellular processes such as autophagy or oxidation of host membranes. Additional studies are required to determine the mechanisms by which ROS can affect GSDMD pores.

## GSDMD-Independent IL-1 Secretion

GSDMD mediates membrane permeability and IL-1 secretion during acute inflammasome signaling (Evavold et al., 2018; Heilig et al., 2018). Permeabilization of the plasma membrane by alternate means is often sufficient to encourage secretion of IL-1 that is independent or secondary to GSDMD. Physical disruption of the membrane is sufficient to mediate IL-1 release secondary to cell lysis after treatment with uric acid crystals (Rashidi et al., 2019). In GSDMD- or caspase-1-deficient cells, long term inflammasome stimulation can lead to IL-1 secretion that is dependent on GSDME or subsequent membrane rupture and likely involves a slow induction of apoptotic signaling (Schneider et al., 2017; Heilig et al., 2020; Zhou and Abbott, 2021). GSDME, like GSDMD, can form membrane pores in both stressed mitochondria and the plasma membrane to facilitate either direct release of IL-1 or the initiation of membrane lysis (Rogers et al., 2017; Wang et al., 2017; Rogers et al., 2019). GSDME requires the activation of apoptotic executioner caspases such as caspase-3/-7 for processing into an N-terminal pore forming fragment (Rogers et al., 2017; Wang et al., 2017). Like GSDMD, GSDME may also promote IL-1α maturation *via* calcium flux and calpain activation (Aizawa et al., 2020). Thus, the primary channels in myeloid cells that mediate IL-1 secretion after inflammasome signaling or caspase-8 activation are GSDMD and GSDME.

GSDME can be activated *in trans* by delivery of granules from CTL and NK cells that contain granzyme B protease (Zhang et al., 2020a). It is conceivable that granzyme-mediated GSDME activation could lead to NLRP3 inflammasome activation through potassium efflux and membrane damage. Other gasdermin family members exist that may show cell type or stimulation specific cleavage and pore forming abilities. Granzyme A may mediate cleavage and activation of GSDMB (Zhou et al., 2020). As this pore could also mediate potassium efflux, granzymes may encourage NLRP3 inflammasome processing and release of IL-1 downstream of GSDMB activation. Therefore, different cell types and stimulations may result in the activation of specific gasdermins allowing for release of IL-1 family members in conjunction with or independent of inflammasomes.

In the context of necroptotic signaling, RIPK3 phosphorylation of the pore forming protein MLKL causes membrane damage that can result in cell lysis. This membrane damage can allow for potassium efflux from the cell leading to activation of the NLRP3 inflammasome (Conos et al., 2017; Gutierrez et al., 2017). NLRP3 activation in this context is required for secretion of bioactive IL-1 primarily through control of IL-1β cleavage, whereas MLKL permeabilization and subsequent lysis is sufficient to allow for passive release independent of GSDMD (Gutierrez et al., 2017). As has been shown for GSDMD, MLKL membrane damage is negatively regulated by ESCRTIII-dependent membrane repair processes (Gong et al., 2017).

Buffering cell culture stimulations or organ explants with the amino acid glycine has been shown to inhibit lysis in response to inflammasome activation and ischemia reperfusion injury (Weinberg et al., 1987; Frank et al., 2000; Fink and Cookson, 2006). While originally thought to discourage osmotic pressure on cell membranes, the discovery of GSDMD and the characterization of the permissive transport of ions and water across the membrane suggest that glycine must inhibit lysis independently of osmotic pressure. Glycine is experimentally used to separate GSDMD pore formation from pyroptotic lysis during inflammasome stimulations (Evavold et al., 2018; Heilig et al., 2018). These experiments illustrated that IL-1β was able to directly traverse GSDMD pores on the membrane and did not require membrane rupture for release. A recent study has discovered that the protein NINJ1 mediates membrane rupture downstream of diverse triggers such as GSDMD pore formation, bacterial toxin pore formation, and late apoptotic signaling that might permit GSDME pore formation (Kayagaki et al., 2021). Notably, MLKL activation during necroptotic signaling appears sufficient to mediate membrane lysis independent of NINJ1. NINJ1-deficient cells provide additional evidence that GSDMD can directly convey IL-1β across the plasma membrane of inflammasome-activated macrophages. Recent work suggests that glycine may impinge upon NINJ1 oligomerization and membrane rupture, though evidence of whether this is a direct effect on NINJ1 or operates on an unknown activation signal of NINJ1 has not been determined (Borges et al., 2021). Thus, glycine buffering and NINJ1 deficiency can be used to chemically and genetically separate IL-1 secretion from lysis in many contexts (Evavold et al., 2018; Heilig et al., 2018; Bjanes et al., 2021; Borges et al., 2021; Kayagaki et al., 2021).

## Transient Membrane Permeability and Hyperactivation

Transient membrane permeability may represent a mechanism of IL-1 secretion (Evavold et al., 2018; Rühl et al., 2018). Recent work has established that cell death and lysis are not necessary consequences of inflammasome activation (Chen et al., 2014; Conos et al., 2016; Gaidt et al., 2016; Wolf et al., 2016; Zanoni et al., 2016). Cells that secrete IL-1 while maintaining energetic viability and resisting membrane rupture are considered hyperactive (Zanoni et al., 2016; Evavold et al., 2018). Certain cell types such as a neutrophils and dendritic cells demonstrate intrinsic resistance to pyroptotic lysis that may represent different membrane reparative capacities, caspase activation dynamics, and expression levels of pore forming proteins such as GSDMD (Chen et al., 2014; Zanoni et al., 2016; Boucher et al., 2018). Some cell types, such as human and porcine blood monocytes, can release IL-1β without cell death as occurs during exogenous treatment of cells with PAMPs such as LPS (Gaidt et al., 2016). However, stimulation of monocytes with combinations of PAMPs can convert a non-lytic release of IL-1β to lytic release in a GSDMD- and ROS-dependent manner (Semino et al., 2018).

An increasing set of stimuli has been reported to induce inflammasome activities and IL-1 release from living (hyperactive) cells (Shimada et al., 2010; Wolf et al., 2016; Zanoni et al., 2016; Evavold et al., 2018). It has been noted that a single inflammasome stimulus can elicit pyroptosis or hyperactivation within the same cell type that presumably depends on the strength of inflammasome signaling (Xia et al., 2021). Cell types may also display varied expression of NINJ1 that result in different thresholds or propensity for cell lysis. This may explain why some cell types such as skin keratinocytes display membrane ballooning after GSDMD and GSDME activation without appreciable cell lysis (Orzalli et al., 2021).

While more mechanistic studies are necessary to define the molecular events that determine inflammasome-dependent activities in dead (pyroptotic) or live (hyperactive) cells, physiological consequences of these activities have proven notable. In particular, the cell fate of hyperactivation has gained attention for its superior ability to activate adaptive immune responses (Zanoni et al., 2016). By adding the IL-1 family to the repertoire of cytokines secreted by activated DCs, modulating hyperactivation has implications for next generation vaccines. Inflammasomes, specifically within hyperactive DCs are able to speed up the differentiation of antigen specific CD8^+^ T cells and the production of long-lived memory T resident memory cells, which are associated with protective immunity in cancer (Zhivaki et al., 2020). In the context of *S. aureus* infection, similar links between cell hyperactivation and protective immunity have been observed (Sanchez et al., 2017). The metabolic profile of hyperactive cells is distinct from naïve or traditionally activated cells, as these cells maintain mitochondrial oxidative phosphorylation while simultaneously utilizing glycolytic activities (Wolf et al., 2016; Zanoni et al., 2016; Di Gioia et al., 2020). In contrast, traditionally activated cells undergo a shift from oxidative phosphorylation to glycolysis. These different metabolic activities and maintenance of mitochondrial polarization may regulate IL-1 secretion, membrane reparative capacity, and cell death (Di Gioia et al., 2020). As the host dynamically regulates metabolism under stress conditions or infection (Pernas, 2021), cells may have evolved alternative methods to secrete IL-1 beyond direct membrane pores in order to retain the threat contextualization of secreted pro-inflammatory IL-1 family members.

## Metabolic Control of IL-1 Secretion

**FIGURE 3:**
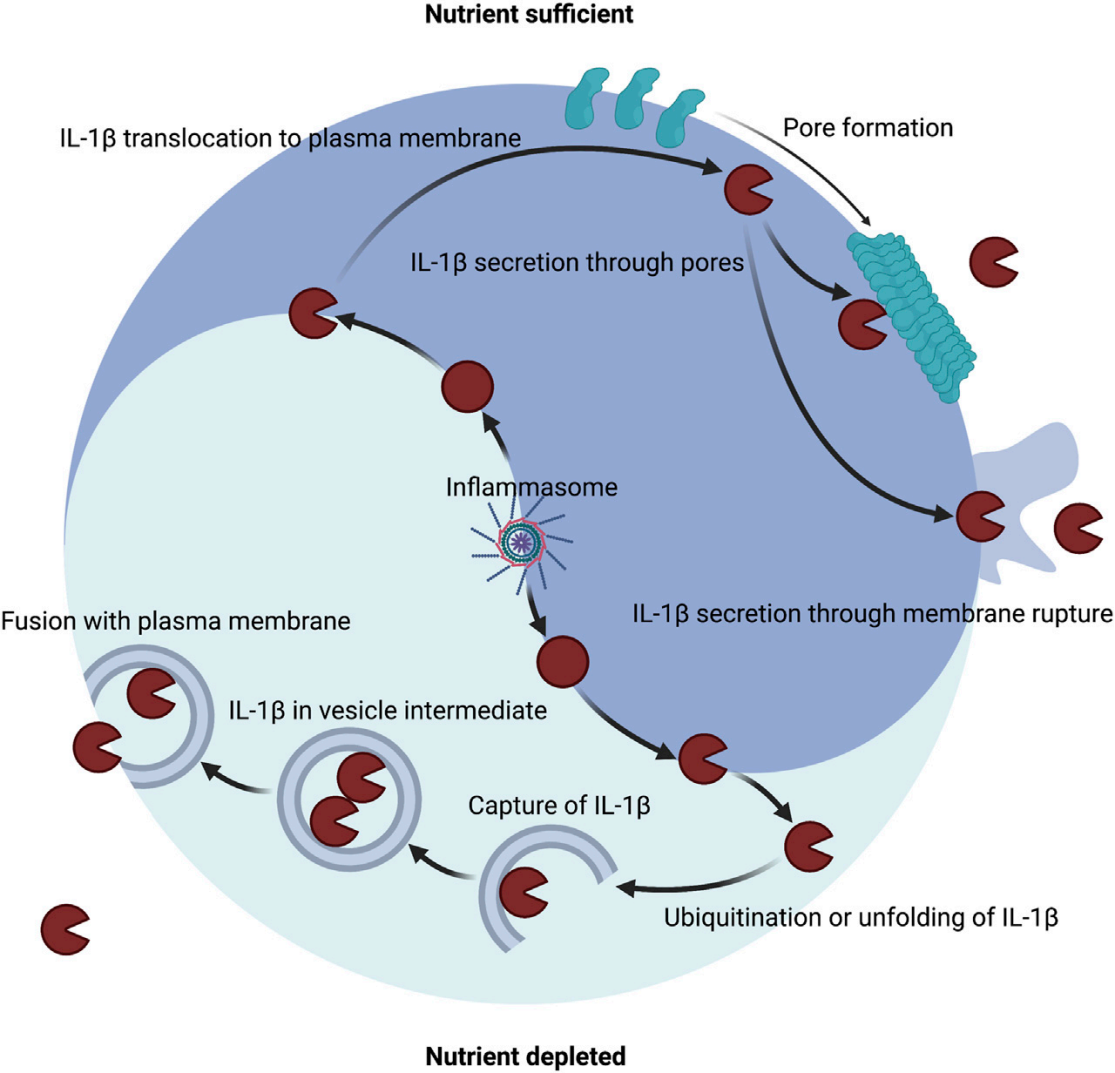
Host metabolic state dictates the route of IL-1β secretion. A major mechanism of IL-1β secretion involves permeabilization of the plasma membrane. Inflammasomes can control the maturation of pro-IL-1β into IL-1β in both nutrient replete or nutrient depleted settings. Inflammasomes can release the pore-forming moiety of GSDMD to encourage membrane permeability for direct secretion and can induce membrane rupture for indirect secretion. Several other membrane permeabilization strategies are sufficient to secrete IL-1β including other gasdermin family members, necroptotic MLKL, bacterial pore forming toxins, and physical disruption. An alternate mechanism of IL-1β secretion may exist that involves capture or translocation into a vesicle intermediate during nutrient depleted or proteotoxic stress settings.

As stated in the prior sections, under nutrient replete conditions a major mechanism of IL-1 secretion is direct conveyance across the plasma membrane through GSDMD pores and other membrane permeabilization strategies (Figure 3). During metabolic dysfunction or starvation, alternative mechanisms may mediate IL-1 secretion (Figure 3). IL-1β can be detected in vesicle intermediates during ER stress and starvation (Dupont et al., 2011; Zhang et al., 2015; Kimura et al., 2017; Zhang et al., 2020b). IL-1β can be ubiquitinated, which may encourage degradation through autophagy and the proteasome or impinge upon cleavage by inflammatory caspases (Harris et al., 2011; Ainscough et al., 2014; Eldridge et al., 2017; Vijayaraj et al., 2021). Autophagy is also known to impinge upon inflammasome signaling (Saitoh et al., 2008; Shi et al., 2012; Liu et al., 2016b). Thus, paradoxically autophagic capture of inflammasomes and cleaved substrates such as of IL-1β may serve as a possible mechanism for increased cellular survival by limiting inflammasome signaling but also promote secretion of low quantities of IL-1β. As such, mature IL-1β may also be secreted *via* autophagic means, but the precise trafficking to prevent degradation has not been determined (Dupont et al., 2011; Kimura et al., 2017). During inflammasome activation, GSDMD may still play a role in autophagic release of IL-1 (Karmakar et al., 2020). In neutrophils, GSDMD is genetically required for IL-1β release (Heilig et al., 2018; Monteleone et al., 2018), but this appears to be independent of plasma membrane localization and pore formation (Karmakar et al., 2020). Instead, GSDMD targets intracellular granules that may allow for IL-1β incorporation into secretory granules (Karmakar et al., 2020). This targeting of secretory granules may allow for a feed forward amplification loop whereby release of granule proteases into the cytosol processes additional IL-1β and GSDMD and calcium flux elicited from the hypercalcemic granules or lysosomes lead to lysosomal exocytosis (Karmakar et al., 2020). Furthermore, deficiency of the autophagy component ATG7 diminished IL-1β in neutrophils suggesting that autophagosomes may also contribute to secretion in addition to perforated secretory granules. Disruption of lysosomes by GSDMD may also explain why autophagosomes may not become degradatory in certain contexts of IL-1β capture.

Ragulator-Rag is purported to control GSDMD pore formation *via* control of GSDMD cleavage during caspase-8 activation in the context of TAK1 inhibition and regulate GSDMD oligomerization through metabolic control of ROS production (Evavold et al., 2021; Zheng et al., 2021). Ragulator-Rag can also mediate repair of endo-membrane damage as evident after treatment with lysosomal damaging agents (Jia et al., 2018; Jia et al., 2020). This may invoke direct activation of macroautophagy as well as indirect upregulation of lysosome biogenesis and autophagy genes downstream of mTOR inactivation and subsequent nuclear translocation of de-phosphorylated TFEB (Sardiello et al., 2009; Settembre et al., 2011; Efeyan et al., 2013; Jia et al., 2018; Jia et al., 2020). Recent work has also suggested that mitochondrial dysfunction is sensed by the Ragulator-Rag complex presumably for autophagic capture of damaged or stressed mitochondria (Condon et al., 2021). Ragulator-Rag may be a general regulator of membrane homeostasis by surveying damaged membranous organelles. Thus, Ragulator-Rag may act to prevent GSDMD-mediated membrane damage in many distinct ways ranging from control of cleavage, oligomerization, and removal of damaged organelles (Jia et al., 2018; Condon et al., 2021; Evavold et al., 2021; Zheng et al., 2021).

Autophagic capture and release of mature IL-1β may operate under diverse metabolic perturbations that could occur in response to stress or microbial invasion (Tattoli et al., 2012; Ravindran et al., 2016). Investigation of whether Ragulator-Rag deficiency, starvation, or other mechanisms of mTOR inhibition decrease GSDMD-mediated IL-1 release while encouraging autophagic means of release are needed. Metabolic perturbations have long been known to affect initiation of cell death signaling through apoptotic, pyroptotic, and necroptotic pathways (Zhang et al., 2009; Andersen and Kornbluth, 2013; Próchnicki and Latz, 2017; Pajuelo et al., 2018). These metabolic perturbations may serve as evolutionary hallmarks of threats to the host such as sterile stressors or pathogenic invasion. Recent studies have identified nuanced metabolic control of terminal stages of death pathways as is evident with control of GSDMD at the stage of cleavage by tricarboxylic acid cycle (TCA) metabolites, oligomerization by ROS metabolites, and pore conformation by phospholipid catabolism (Humphries et al., 2020; Bambouskova et al., 2021; Evavold et al., 2021; Santa Cruz Garcia et al., 2022). As microbes may also have evolved mechanisms to manipulate these endogenous metabolic checkpoints, alternative mechanisms of IL-1 release are crucial to convey threat levels to other cells. IL-1 family cytokines can poise or prime cells for cell-intrinsic immunity or detection of higher-level threats (Garlanda et al., 2013; Evavold and Kagan, 2019). IL-1 family cytokines can also encourage local inflammation and recruitment of additional innate and adaptive leukocytes (Garlanda et al., 2013). In addition, IL-1 family cytokines can reprogram organismal metabolism through fever (Garlanda et al., 2013). The intersection between host defense and metabolism is a burgeoning area of investigation. Studies on IL-1 family cytokines as both initiators and responders to host metabolic state are sure to follow.

## Concluding Remarks and Outstanding Questions

Of the IL-1 family members, IL-1α and IL-1β have been the most characterized in terms of bioactivity, activation, and secretion. Based on current evidence for the multi-step regulation of these prototypical nuclear and cytosolic IL-1 family members, we speculate that similar mechanisms may exist for the activation and secretion of other leaderless IL-1 family members. Specifically, we predict that the crossing of topological barriers, such as the nuclear and/or plasma membranes, represents a point of regulation for other IL-1 family members. Whereas nuclear IL-1 family members may be constitutively expressed yet confined by an added physical barrier, cytosolic IL-1 family members may be primarily regulated by context-dependent transcription and refined proteolytic cleavage by the secreting cell or other cell types. Further studies are required to determine the signals that instruct the nuclear release of IL-1α, IL-33, and IL-37, and in the case of newly synthesized membrane-bound IL-1α, more work is required to delineate the mechanisms that instruct the trafficking to and crossing of the plasma membrane. Perhaps due to inflammatory nature of secreted IL-1 family cytokines, compensatory mechanisms regulate IL-1 proteins post-secretion. For example, decoy receptors, binding proteins, and inactive IL-1 family structural analogues (termed receptor antagonists) further buffer the bioactivity of the IL-1 family in the extracellular space. These buffering systems likely exist to limit inflammation in the context of homeostatic death processes and may be upregulated during the resolution phase of inflammation. Moreover, metabolic control of IL-1 family cytokines likely constitutes another pathway of regulation. While intact metabolism may primarily affect IL-1β secretion *via* encouraging membrane permeability, alternate routes of secretion may occur in nutrient deplete contexts or proteotoxic stress. For instance, translocation or capture of IL-1β into vesicle intermediates may rely on the metabolic status of the cell. Analogous metabolic mechanisms may also exist in the regulation of other IL-1 family members. In terms of membrane permeabilization strategies employed by the host to secrete IL-1, GSDMD and GSDME are the best characterized. Recent studies have begun to discover host or pathogen driven activation programs for other gasdermin family members. Additional studies have implicated distinct mechanisms of membrane permeabilization or rupture mediated through MLKL, NINJ1, and bacterial pore forming toxins as well as physical disruption as being sufficient for mediating release of IL-1. Cell type specific or pathogen specific programs may therefore exist that mediate the secretion of particular IL-1 family cytokines in response to unique membrane permeabilization strategies.
